# Acute and subchronic toxicity of aqueous extracts of *Combretum micranthum* (G. Don) and *Gardenia sokotensis* (Hutch) having ethnobotanical uses in Burkina Faso

**DOI:** 10.1016/j.toxrep.2025.102097

**Published:** 2025-07-28

**Authors:** OUEDRAOGO Elisabeth, ZABRE Généviève, TINDANO Basile, YOUGBARE Wendyam Joëlle Raymonde, OWONA Pascal Emmanuel, BAYALA Balé

**Affiliations:** a Laboratory of Animal physiology, UFR/SVT, Université Joseph KI-ZERBO, Ouagadougou BP 7021, Burkina Faso; b Laboratory of Animal physiology, Faculty of sciences, University of Yaoundé 1, Yaoundé P.O. 812, Cameroon

**Keywords:** Acute toxicity, Combretum micranthum, Gardenia sokotensis, Ethnobotanical survey, Phytochemical screening, Subchronic toxicity

## Abstract

Medicinal plants are the major sources of drugs used to treat diseases. Scientific studies were performed on some plants, but few data are available on the medicinal plants used to manage bone diseases in Burkina Faso. This study was conducted to identify medicinal plants used in the treatment of osteoporosis and investigate the acute and subchronic toxicity of *Combretum micranthum* and *Gardenia sokotensis* aqueous extracts. A survey was carried out through a structured interview with traditional practitioners. Phytochemical screening was performed using a validated thin-layer chromatographic method. The acute oral toxicity study of extracts was validated at 2000 mg/kg in mice. In the subchronic toxicity, rats were orally administered 100, 200, and 400 mg/kg of each extract for 90 days. Results show sixty-one plant species divided into 33 families. *C. micranthum* and *G. sokotensis* were most cited. Phytochemical screening of aqueous extracts of plants revealed flavonoids, tannins, and terpenoids. Acute toxicity study indicated up to 2000 mg/kg of each extract was tolerated without death or any signs of toxicity. In the subchronic toxicity test, physiological, serum biochemistry, and hematology examination, no features suggestive of each extract's toxicity were observed at doses of 100 and 200 mg/kg. The hepatic balance (aspartate and alanine aminotransferases) was significantly (p < 0.0001) reduced at doses of 100 and 200 mg/kg. A significant (p < 0.001) decrease in triglyceride and cholesterol levels was observed. To conclude, extracts were non-toxic and could be used for their ethnopharmacological properties, but experimental therapeutic evidence is still needed.

## Highlights

• *Combretum micranthum* and *Gardenia sokotensis* are hepatoprotective and nephroprotective.
• *Combretum micranthum* and *Gardenia sokotensis* present a hepatic risk at high doses.
• *Combretum micranthum* and *Gardenia sokotensis* reduce triglyceride levels.

## Graphical Abstract

**Figure:**
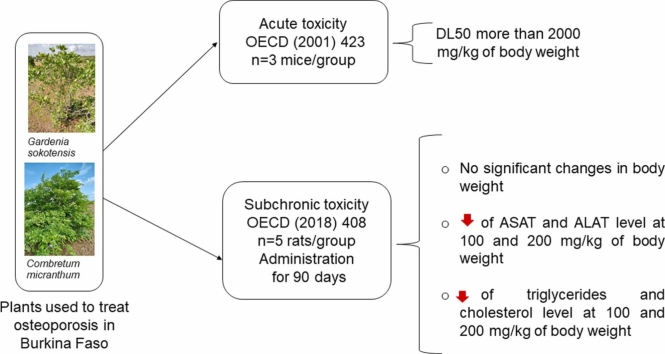

## 1 Introduction

Medicinal plants have been used for centuries to treat many illnesses and conditions [1]. According to the World Health Organization, 80 % of the world’s population depends on phytotherapy and the use of medicinal plants to meet health needs. Traditional African medicine is the oldest and perhaps the most varied of medical systems [2]. In traditional African medicine, the *Combretum* and *Gardenia* genera are highly valued [3], [4]. More than 130 species of the Gardenia genus are distributed throughout the tropical regions of Asia, Africa, Australia, Madagascar, and certain Pacific Islands [5] and about 370 species of the *Combretum* genus [6]. Gardenia genus has many properties through its components. *Gardenia jaminoides* has immunomodulatory properties [7]. The triterpenoids and saponins confer antibacterial properties to *Gardenia ternifolia* [8]. *Gardenia sootepensis* has anti-inflammatory properties [9]. *Gardenia gummifera* methanolic extract has an antioxidant and hepatoprotective effect [10]. The roots of *Gardenia sokotensis* have shown anti-trypanosomiasis properties [11]. Its leaves have cytotoxic activity against cancer cells [12]. *Gardenia sokotensis* is used as a muscle toner [13]. Its seeds and pulp contain an appreciable amount of nutrients and secondary metabolites that could serve as an additional source of food and medicine [14]. In Mali, *Gardenia sokotensis* is used to fight malaria [15]. Pharmacological studies have shown various properties of the *Combretum* genus: anti-inflammatory, antifungal, antibacterial, and anti-diabetic [16], [17]. *Combretum erythrophyllum* has great medicinal importance; its roots and leaves are used to treat venereal diseases and abdominal pain, while its bark is known to treat leprosy and wounds [18]. *C. erythrophyllum* contains secondary metabolites with anti-inflammatory, anti-bacterial, and antioxidant properties [19]. In Burkina Faso, the availability and efficacy of plants, and above all the low cost of medicinal recipes, make traditional pharmacopoeia the main source of healthcare for an estimated 70 % of the population [20]. The flora of Burkina Faso includes a diversity of plants used to treat diseases. However, it is important to note that some medicinal plants can be toxic if consumed in large quantities. The toxicity of plants or their compounds can lead to cytotoxicity. This study aims to make a scientific contribution to the toxicity studies of *C. micranthum* and *G. sokotensis*, two medicinal plants of traditional medicine in Burkina Faso.

## 2 Material and methods

### 2.1 Material

#### 2.1.1 Study area

The study was carried out in three regions of Burkina Faso. The Centre region in Ouagadougou city, with a population of 3030,384 is located in the Sahelian-Sudanese zone at 12°20' north latitude and 1°30' east longitude. The Haut-Bassins region in Bobo-Dioulasso city, with a population of 2239,840, is located at 11° 11' 00'' north 4° 17' 00'' west. The East-Center region in Tenkodogo city is located at 11° 46' 59'' North and 0° 21' 57'' West with a population of 1580,508 [21].

#### 2.1.2 Plant material

*Combretum micranthum* and *Gardenia sokotensis* leaves were collected in Sabcé, 98 km from Ouagadougou (Burkina Faso) between 6:00 and 11:00 GMT. The leaves were thoroughly washed with water and dried in a ventilated area away from sunlight and dust. The samples of each plant were collected, identified and deposited in the herbarium of Université Joseph KI-ZERBO (Burkina Faso) where they were registered under the voucher numbers 7002 for *G. sokotensis* and 7003 for *C. micranthum.*

#### 2.1.3 Test organism

Female Wistar rats and female mice NMRI supplied by the Université Joseph KI-ZERBO animal facility, were used for the different experiments. Every effort was made to minimize animal suffering and reduce the number of animals used. The animals were housed in groups of 3 and 5 per cage, respectively for the acute and the subchronic toxicity studies under standard husbandry conditions, including a temperature of 22° ± 3°C, relative humidity of 50 ± 10 % and a photo-period (12 h/12 h, light/dark cycle). The animals were fed with rodent pellets and had free access to tap water. All experiments were performed following the guidelines of the Université Joseph KI-ZERBO Animal Protection Ethics Committee (CE-UJKZ/2023–05).

### 2.2 Methods

#### 2.2.1 Ethnobotanical survey

The ethnobotanical survey was conducted from June to December 2022 through structured interviews. Pre-structured questionnaires were used for interviewing representatives of the traditional practitioners. First, an interview was conducted with representatives of the traditional practitioners in each locality. The aim was to explain the objectives of the study. The objectives of the study were explained to each traditional healer met, to obtain their consent to participate in the study. The interview was conducted in the language of the traditional healer’s choice. The use of an interpreter was considered when necessary.

#### 2.2.2 Extracts preparation

The extraction was carried out using medicinal and aromatic plant extraction technologies [22]. Briefly, the dry leaves of each species were pulverized using a mechanical grinder. Extraction was carried out from the powder obtained. Five hundred grams (500 g) of powder of each species were added to a beaker containing 1500 mL of distilled water. After homogenization, the mixture was boiled for 30 min. After cooling, the decoction was filtered and centrifuged at 2000 revolutions per minute for 10 min. The supernatant was frozen and lyophilized. Aqueous extracts of *Combretum micranthum* (CMAE) and *Gardenia sokotensis* (GSAE) were collected and stored at 4°C for the experiments.

#### 2.2.3 Phytochemical screening

Phytochemical screening of extracts was carried out on TLC (10cm×10cm) silica gel plates ${60F}_{254}$ Merck, Darmstadt, Germany [23], [24]. Five microliters (5 µL) of extract (concentration = 20 mg/mL) were deposited in a 0.8 cm strip with a semi-automatic sample dispenser (CAMAG, Linomat 5, Switzerland) along the baseline 8 mm from the lower edge of the plate. The distance between spots was 3.4 mm. The distance between the plate’s first spot and left edge, and between the last spot and the right edge was 20 mm. A constant application rate of 100 nL/s was used. Linear ascending development with 10 mL mobile phase was carried out in a CAMAG double-trough glass chamber lined with filter paper and saturated with mobile phase vapour for 20 min. The development distance was approximately 70 mm. The plates were dried after development using a hair dryer. In the double-trough chamber, the mobile phases were [25]:

- Flavonoids, tannins, and saponosides: ethyl acetate/formic acid/water (80: 10: 10, v/v/v). Flavonoids were revealed by Neu's reagent, tannins by FeCl_3_ (2 %), and saponosides by sulfuric anisaldehyde.

- Terpenoids: ethyl acetate /n-hexane (20: 4, v/v). Terpenoids were revealed by the Liebermann Bürchard reagent.

#### 2.2.4 Acute toxicity assessment

The acute toxicity test was performed according to OECD guideline number 423 [26]. Nine (09) nulliparous female mice of 8 weeks old with a mean weight of 29 ± 2.44 g were used. They were divided into 3 groups of 3 mice each. Four (4) hours before the administration of the extracts, the mice were deprived of water. Group 1 served as a control and received distilled water. A single dose of 2000 mg/kg body weight (bw) of CMAE and GSAE was administered orally to groups 2 and 3 respectively. Animals were first observed for one hour after treatment, before receiving food and water. Observations continued for 24 h, 48 h, 72 h, and daily during 14 days after treatment. Parameters such as mortality, mobility, food and water consumption, eye colour, convulsions, drowsiness, type of defecation (diarrhea or not), lethargy, and salivation were recorded.

#### 2.2.5 Subchronic toxicity assessment

The subchronic toxicity study was performed according to OECD guideline number 408 [27]. Fifty (50) nulliparous, non-pregnant, two-month-old female Wistar rats (considering higher sensitive than males), weighing an average of 100 ± 0.90 g, were used and divided into 10 groups of 5 animals each. Groups 1 and 2 served as control and satellite control. They were gavaged with distilled water. Groups 3, 4, 5, 6, 7, and 8 were treated with aqueous extracts of *Combretum micranthum* and *Gardenia sokotensis* at different doses: 100, 200, and 400 mg/kg bw of each extract. Groups 9 and 10 served as satellites and were treated with dose of 400 mg/kg bw for each plant. The treatments were administered orally, daily for 90 consecutive days. Rats were weighed once a week during the experimental period to determine body weight gain. Satellite groups were observed for two weeks after cessation of treatments to assess reversibility, persistence, or delayed signs of toxic effects.

#### 2.2.6 Animal sacrifice and sample preparation

At the end of the experiment, animals were made water-free and fasted for 12 h. They were then anaesthetized using ketamine/lidocaine (1/0.7). Blood was collected using dry tubes and EDTA (ethylene diamine tetra acetate) tubes. Blood from dry tubes was centrifuged at 3000 revolutions per minute for 15 min. The resulting serum was used for biochemical analysis using a spectrophotometer. The blood from EDTA tubes was used for hematological studies. The rats were dissected, and organs such as the heart, liver, kidneys, and spleen were collected and weighed for the assessment of relative organ weights according to the formula [28]:

Wr = (wo/wb) X 100

Where W*r* is the relative organ weight, wo is the organ weight (g), and wb is the rat body weight (g).

#### 2.2.7 Biochemical analysis

Parameters such as total cholesterol, triglyceride concentrations, urea, direct and total bilirubin levels in serum were determined using commercial diagnostic (Atlas Medical, Germany) by the colorimetric method of Jaffé [28]. The concentrations of creatinine and activities of alanine and aspartate aminotransferases (ALT and AST) were also determined spectrophotometrically using commercial diagnostic kits (Atlas Medical, Germany) by the kinetic method, UV-IFCC optimized.

#### 2.2.8 Data analysis

The plants were identified using their local names and compared to literature [29], [30]. GraphPad Prism software version 9.5.1 was used for graphing and statistical analysis using One-way analysis of variance (ANOVA I) followed by Tukey’ test as a post-hoc analysis. The value of p < 0.05 was considered to be statistically significant. The frequency of citation (F) of each species was calculated to determine which species is more valuable according to traditional practitioners. It was calculated using the following formula [31]:

**F = number of responses citing species x 100/ total number of quotes**

The Informant Consensus Factor (ICF) was calculated to assess the agreement of information on the use of plants for osteoporosis and related diseases [31].

**ICF = Na/Nt,** where **Na** is the number of informants citing a species; **Nt** is the total number of informants. The ICF ranges from 0 to 1. A low value, close to 0, indicates that informants disagree about the proposed therapies. When this value is close to 1, it indicates a high level of agreement on the use of the plant.

## 3 Results

### 3.1 Ethnobotanical survey

#### 3.1.1 Traditional practitioners' profile

**Fig. 1:**
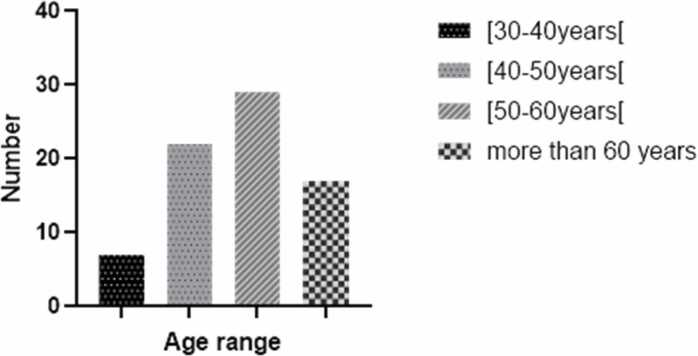
Distribution of traditional practitioners based on their age.

The survey results showed a predominance of men (57 %) compared to women (43 %). Traditional practitioners ranged in age from 30 to 75, with the majority being at least 50 years old (Fig. 1).

**Fig. 2:**
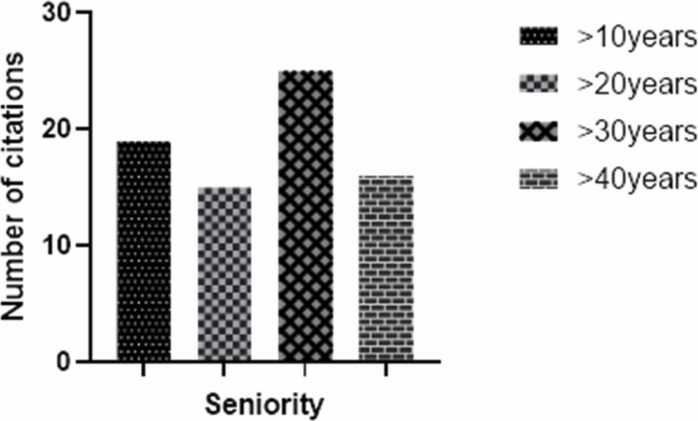
Distribution of traditional practitioners based on their seniority.

All the traditional practitioners surveyed have at least 10 years’ experience in their profession (Fig. 2), with 33 % having at least 30 years’ experience.

#### 3.1.2 Plants identified

At the end of the survey, 61 species of plants were cited by 75 traditional practitioners to manage osteoporosis and related diseases (Table I). These species of plants are divided into 23 orders and 33 families. The Fabaceae family included 14 genera, and the Rubiaceae, Moraceae, and Combretaceae families each included 3 genera. *Gardenia sokotensis*, from the Rubiaceae family and the Gentianales order, is the most cited species with a frequency of 12 %, followed by *Combretum micranthum* from the Combretaceae family and the Myrtales order with a frequency of 9.33 %.

**Table I:** Plant species and their citation frequencies.

**Species**	**Order**	**Family**	**Biological types**	**Citation**	**consensus usage factor**
				**frequencies (%)**	
*Hygrophila auriculata*	Lamiales	Acanthaceae	herb	0.67	0.01
*Lannea acida*	Sapindales	Anacardiaceae	tree	2.00	0.04
*Lannea macrocarpa*	Sapindales	Anacardiaceae	tree	0.67	0.01
*Annona senegalensis*	Magnoliales	Annonaceae	tree	1.33	0.03
*Saba senengalensis*	Gentianales	Apocynaceae	liana	2.00	0.04
*Chrisantellum americanum*	Asterales	Asteraceae	herb	0.67	0.01
*Chrysanthellum indicum*	Asterales	Asteraceae	herb	0.67	0.01
*Caloptropis procera*	Gentianales	Asclepiadaceae	shrub	2.00	0.04
*Adansonia digitata*	Malvales	Bombacaceae	tree	1.33	0.03
*Cordia mixa*	Boraginales	Boraginaceae	tree	0.67	0.01
*Crescentia cujete*	Scrophulariales	Bignoniaceae	tree	0.67	0.01
*Boscia senegalensis*	Brassicales	Capparaceae	shrub	0.67	0.01
*Maerua angolensis*	Brassicales	Capparaceae	tree	0.67	0.01
*Cassia sieberiana*	Fabales	Caesalpiniaceae	shrub	2.67	0.05
*Tamarindus indica*	Fabales	Caesalpiniaceae	tree	8.00	0.16
*Maytenus senegalensis*	Celastrales	Celastraceae	tree	0.67	0.01
*Cochlospermum tinctorium*	Malvales	Cochlospermum	liana	0.67	0.01
*Anogeisus leiocarpa*	Myrtales	Combretaceae	tree	0.67	0.01
*Combretum micranthum*	Myrtales	Combretaceae	shrub	9.33	0.19
*Guiera senegalensis*	Myrtales	Combretaceae	shrub	7.33	0.15
*Ipomoea asarifolia*	Solanales	Convolvulaceae	herb	0.67	0.01
*Euphobia hirta*	Euphorbiales	Euphorbiaceae	herb	0.67	0.01
*Acacia ataxacantha*	Fabales	Fabaceae	tree	0.67	0.0
*Afrormosia laxiflora*	Fabales	Fabaceae	tree	0.67	0.01
*Bauhinia rufescens*	Fabales	Fabaceae	shrub	0.67	0.01
*Crotalaria naragutensis*	Fabales	Fabaceae	herb	0.67	0.01
*Crotalaria pallida*	Fabales	Fabaceae	herb	0.67	0.01
*Detarium microcarpum*	Fabales	Fabaceae	tree	4.67	0.09
*Dichrostachys glomerata*	Fabales	Fabaceae	tree	0.67	0.01
*Pericopsis laxiflora*	Fabales	Fabaceae	tree	0.67	0.01
*Mimosa pigra*	Fabales	Fabaceae	tree	0.67	0.01
*Piliostigma reticulatum*	Fabales	Fabaceae	shrub	2.00	0.04
*Piliostigma thonningii*	Fabales	Fabaceae	shrub	0.67	0.01
*Prosopis africana*	Fabales	Fabaceae	tree	0.67	0.01
*Pterocarpus erinaceus*	Fabales	Fabaceae	tree	0.67	0.01
*Senna occidentalis*	Fabales	Fabaceae	shrub	0.67	0.01
*Mentha ssp*	Lamiales	Lamiaceae	herb	0.67	0.01
*Ocimum basilicum*	Lamiales	Lamiaceae	herb	0.67	0.01
*Khaya senengalensis*	Sapindales	Meliaceae	tree	2.67	0.05
*Trichilia emitica*	Sapindales	Meliaceae	tree	0.67	0.01
*Entada africana*	Fabales	Mimosaceae	tree	0.67	0.01
*Parkia biglobosa*	Fabales	Mimosaceae	tree	0.67	0.01
*Ficus ingens*	Urticales	Moraceae	tree	2.00	0.04
*Ficus iteophylla*	Rosales	Moraceae	tree	0.67	0.01
*Ficus sycomorus*	Rosales	Moraceae	tree	0.67	0.01
*Moringa oleifera*	Brassicales	Moringaceae	tree	2.67	0.05
*Ximenia americana*	Santalales	Olacaceae	shrub	2.00	0.04
*Opilia amentacea*	Santalales	Opiliacea	tree	0.67	0.01
*Hymenocardia acida*	Malpighiales	Phyllanthaceae	tree	0.67	0.01
*Phyllanthus amarus*	Malpighiales	Phyllanthaceae	herb	0.67	0.01
*Bambusa vulgaris*	Poales	Poaceae	herb	0.67	0.01
*Zea mays*	Poales	Poaceae	herb	0.67	0.01
*Crossopteryx febrifuga*	Gentianales	Rubiaceae	tree	0.67	0.01
*Feretia apodanthera*	Gentianales	Rubiaceae	shrub	1.33	0.03
*Gardenia erubensces*	Gentianales	Rubiaceae	shrub	2.00	0.04
*Gardenia sokotensis*	Gentianales	Rubiaceae	shrub	12.00	0.24
*Fagara xanthoxyloides*	Sapindales	Rutaceae	shrub	1.33	0.03
*Paullinia pinnata*	Sapindales	Sapindaceae	liana	0.67	0.01
*Vitellaria paradoxa*	Ericales	Sapotaceae	tree	2.67	0.05
*Talinum triangulare*	Caryophyllales	Talinaceae	herb	0.67	0.01
*Aframomum melegueta*	Zingiberales	Zingiberaceae	herb	1.33	0.03
*Balanites aegyptiaca*	Sapindales	Zygophyllaceae	tree	0.67	0.01
**Total number of citations**	**150**				
**Total number of informers**	**75**				

#### 3.1.3 Plant parts used and treatment

**Fig. 3:**
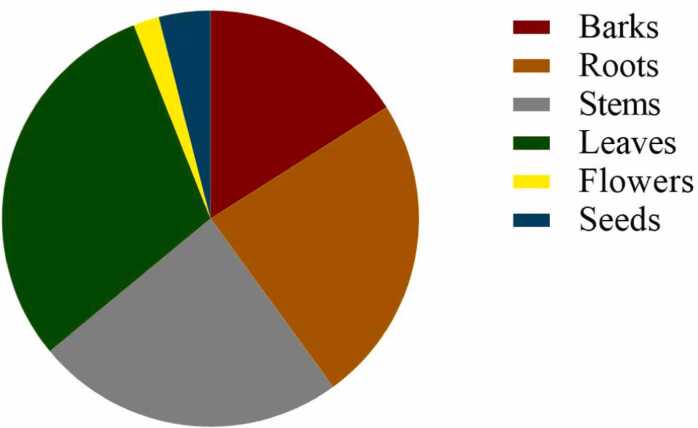
Distribution of the different parts of the plants used.

All parts of the plant mentioned are used to treat osteoporosis and related conditions. Leaves are the most widely used (30 %). The main preparation method was decoction. Underground parts are used less frequently than above-ground parts (Fig. 3).

### 3.2 Plants compounds analysis

#### 3.2.1 Phytochemical screening

Phytochemical screening was employed to identify the components of *C. micranthum* and *G. sokotensis* aqueous extracts. It revealed the presence of flavonoids, saponosides, tannins and terpenoids (Table II).

**Table II:** Phytochemical compounds of C. micranthum and G. sokotensis aqueous extracts.

**Samples**	***C. micranthum***	***G. sokotensis***
**TPC** (mg of GAE/g)	103.79 ± 0.07	111.81 ± 0.21
**TFC (**mg of QE/g)	11.50 ± 0.02	33.80 ± 0.49
**Saponosides** (mg of DE/g)	6.83 ± 0.13	6.81 ± 0.12

TPC: total phenolic compounds; TFC: total flavonoid content; GAE: gallic acid equivalent; QE: quercetin equivalent; DE: diosgenin equivalent.

### 3.3 Acute toxicity

Oral administration of *C. micranthum* (CMAE) and *G. sokotensis* (GSAE) aqueous extracts resulted in no mortality at the dose of 2000 mg/kg bw compared to the control animals and did not induce any apparent signs of toxicity. The extracts did not cause any visible changes in all the investigated signs of toxicity as compared to the normal control group. No difference in behaviour was observed during the 14-day observation period compared with the first few hours after administration.

### 3.4 Subchronic toxicity

#### 3.4.1 Effect of *C. micranthum* and *G. sokotensis* aqueous extracts on weight gain

**Fig. 4:**
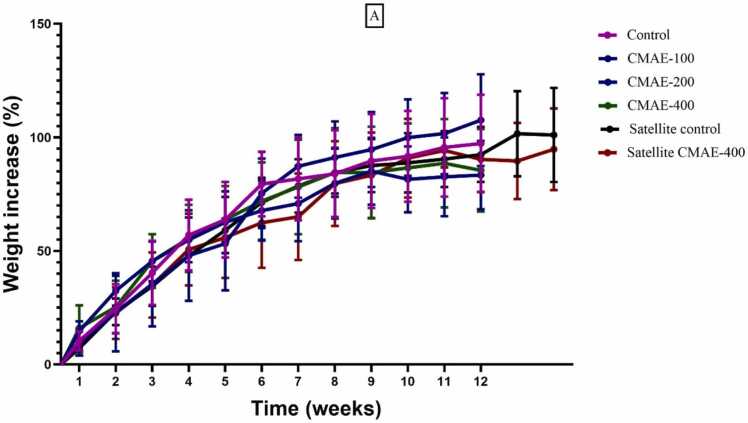

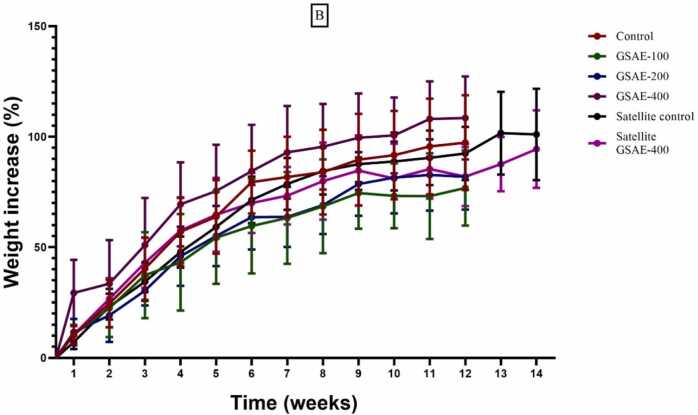
**A.** Trend of body weight changes in each group after taking C. micranthum aqueous extract for 90 days and during a 14-day recovery period. Data are expressed as Mean ± SEM (n = 5), CMAE: rats treated with C. micranthum aqueous extract at doses of 100, 200, and 400 mg/kg.; B. Trend of body weight changes in each group after taking G. sokotensis aqueous extract for 90 days and during a 14-day recovery period. Data are expressed as Mean ± SEM (n = 5), GSAE rats treated with aqueous extract of G. sokotensis at doses of 100, 200, and 400 mg/kg;.

During the 90-day treatment period, both control and treated groups appeared to be in good health. No signs of lethargy or mortality were observed during this period. Animals in the CMAE-treated and GSAE-treated groups, as well as those in the control group, increased in body weight gain during this period (Fig. 4).

#### 3.4.2 Effect of *C. micranthum* and *G. sokotensis* aqueous extracts on the relative weight of organs

Macroscopic observations showed no change in organ color or texture. Administration of CMAE did not result in any significant (p > 0.05) change in heart, liver, spleen, or kidney weights compared to the control group (Table III).

**Table III:** Effect of C. micranthum aqueous extract on the relative weight of organs.

	Heart	Liver	Spleen	Kidney
Control	0.38 ± 0.07	2.92 ± 0.17	0.27 ± 0.02	0.54 ± 0.02
CMAE 100 mg/kg	0.40 ± 0.03	3.23 ± 0.23	0.32 ± 0.13	0.56 ± 0.02
CMAE 200 mg/kg	0.44 ± 0.08	3.29 ± 0.21	0.27 ± 0.03	0.57 ± 0.10
CMAE 400 mg/kg	0.38 ± 0.02	2.57 ± 0.16	0.24 ± 0.03	0.55 ± 0.03
Satellite control	0.42 ± 0.08	3.00 ± 0.14	0.28 ± 0.06	0.54 ± 0.02
Satellite 400 mg/kg	0.45 ± 0.08	3.18 ± 0.35	0.32 ± 0.11	0.56 ± 0.02

Changes in the relative weight of organs after taking *C. micranthum* aqueous extract (CMAE) for 90 days and during 14-day recovery period. Data are expressed as Mean ± SEM (n = 5), p > 0.05.

Similarly, no significant (p > 0.05) differences in relative weight of organs (heart, liver, spleen and kidney) were observed between control and treated groups after GSAE administration (Table IV).

**Table IV:** Effects of G. sokotensis aqueous extract on relative weight of organs.

	Heart	Liver	Spleen	Kidney
Control	0.38 ± 0.07	2.92 ± 0.17	0.27 ± 0.02	0.54 ± 0.02
GSAE 100 mg/kg	0.41 ± 0.03	2.88 ± 0.07	0.24 ± 0.05	0.54 ± 0.03
GSAE 200 mg/kg	0.41 ± 0.03	2.90 ± 0.05	0.23 ± 0.01	0.53 ± 0.02
GSAE 400 mg/kg	0.45 ± 0.01	2.87 ± 0.01	0.23 ± 0.00	0.54 ± 0.02
Satellite control	0.42 ± 0.08	3.00 ± 0.14	0.28 ± 0.06	0.54 ± 0.02
Satellite 400 mg/kg	0.42 ± 0.03	3.03 ± 0.11	0.28 ± 0.01	0.56 ± 0.02

Changes in the relative weight of organs after taking *G. sokotensis* aqueous extract (GSAE) for 90 days and during 14-day recovery period. Data are expressed as Mean ± SEM (n = 5), p > 0.05.

#### 3.4.3 Effect of *C. micranthum* and *G. sokotensis* aqueous extracts on liver biomarkers

Blood levels of alanine aminotransferase (ALAT) and aspartate aminotransferase (ASAT) were significantly (p < 0.000 l) decreased at doses of 100 and 200 mg/kg bw for each extract compared to the control. These levels increased significantly (p < 0.000 l) at the dose of 400 mg/kg bw. On the other hand, after 14 days of observation without extract administration, there was no significant (p > 0.05) variation between satellites. The aqueous extract of *C. micranthum* at the doses of 200 and 400 mg/kg showed a very significant increase (p < 0.00 l) in total bilirubin levels compared to the control. In contrast, at the dose of 100 mg/kg, the extract produced a non-significant (p > 0.05) decrease in total bilirubin levels compared to the control. This non-significant (p > 0.05) decrease was observed at all doses of extract for direct bilirubin levels. Total bilirubin levels in the groups treated with *G. sokotensis* extract increased very significantly (p < 0.00 l) compared to the control group (Table V). The change in direct bilirubin was not significant (p > 0.05).

**Table V:** Effect of C. micranthum and G. sokotensis aqueous extracts on liver biomarkers.

	ALAT(UI/L)	ASAT(UI/l)	Total bilirubin (mg/L)	Direct bilirubin (mg/L)
Control	87.6 ± 2.3	7.6 ± 0.5	31.4 ± 0.9	27.7 ± 1.1
CMAE 100 mg/kg	19.8 ± 1.8^***^	5.2 ± 0.8^***^	28.7 ± 1.4	19.5 ± 1.5
GSAE 100 mg/kg	42.8 ± 0.8^***^	5.6 ± 0.4^***^	53.7 ± 2.9^***^	20.9 ± 2.2
CMAE 200 mg/kg	74.0 ± 1.6^***^	5.8 ± 0.8^**^	61.2 ± 2.1^***^	26.7 ± 1.9
GSAE 200 mg/kg	50.0 ± 1.6^***^	7.4 ± 0.5	51.9 ± 2.6^***^	24.9 ± 3.5
CMAE 400 mg/kg	103.8 ± 1.9^***^	9.4 ± 0.9^***^	78.2 ± 1.0^***^	27.8 ± 1.4
GSAE 400 mg/kg	96.4 ± 0.5^***^	11.6 ± 0.5^***^	55.0 ± 1.8^***^	22.9 ± 0.9
Satellite control	93.0 ± 1.6	10.8 ± 0.4	36.7 ± 2.1	25.1 ± 1.5
Satellite CMAE 400 mg/kg	96.8 ± 1.5^**#**^	9.4 ± 0.5^**#**^	43.4 ± 1.1^**###**^	27.8 ± 1.4
Satellite GSAE 400 mg/kg	91.0 ± 1.0	10.6 ± 0.5	34.0 ± 0.2	23.8 ± 0.5

Alanine aminotransferase (ALAT), Aspartate aminotransferase (ASAT), biochemical parameters measured in serum after 90 days of C. micranthum (CMAE) and G. sokotensis (GSAE) aqueous extracts administration. Data are expressed as Mean ± SEM (n = 5), ** p < 0.001, *** p = 0.0001, compared to the control group. # p < 0.05, ## p < 0.001, ### p = 0.0001, compared to the satellite control group.

#### 3.4.4 Effect of *C. micranthum* and *G. sokotensis* aqueous extracts on kidney biomarkers

Extracts at all doses resulted in a significant (p < 0.0001) reduction in the creatinine level compared to the control. At all doses, the extracts did not cause any significant variation in urea levels compared to the control (Table VI).

**Table VI:** Effect of *C. micranthum* and *G. sokotensis* aqueous extracts on kidney biomarkers.

	Creatinine (mg/dL)	Urea (mg/dL)
Control	0.87 ± 0.03	46.67 ± 5.47
CMAE 100 mg/kg	0.82 ± 0,01	46.68 ± 5.47
GSAE 100 mg/kg	0.74 ± 0.01	46.67 ± 7.07
CMAE 200 mg/kg	0.78 ± 0.01	46.67 ± 1.14
GSAE 200 mg/kg	0.74 ± 0.00	46.66 ± 1.34
CMAE 400 mg/kg	0.80 ± 0.01	46.68 ± 4.47
GSAE 400 mg/kg	0.83 ± 0.01	46.67 ± 8.36
Satellite control	0.87 ± 0.03	46.67 ± 8.36
Satellite CMAE 400 mg/kg	0.88 ± 0.01	46.68 ± 0.00
Satellite GSAE 400 mg/kg	0.88 ± 0.01	46.67 ± 5.47

Creatinine and urea biochemical parameters measured in serum after 90 days of C. micranthum (CMAE) and G. sokotensis (GSAE) aqueous extracts administration. Data are expressed as Mean ± SEM (n = 5), p > 0.05, compared with the control group.

#### 3.4.5 Effect of *C. micranthum* and *G. sokotensis* aqueous extracts on triglyceride, cholesterol, and blood glucose

The *C. micranthum* and *G. sokotensis* aqueous extracts at doses of 100 mg/kg bw produced a non-significant (p > 0.05) decrease in triglyceride level compared to the control. Cholesterol level in the treated groups with extracts decreased very significantly (p < 0.00 l) compared to the control group. Except the dose of 100 mg/kg bw, where this variation was not significant (p > 0.05), triglyceride level in the treated groups decreased very significantly (p < 0.00 l) compared to the control group. Blood glucose level was highly significantly (p < 0.000 l) lower than control, except the dose of 400 mg/kg, where level was highly (p < 0.000 l) increased compared with control. There was no significant (p > 0.05) variation between satellite groups (Table VII).

**Table VII:** Effect of C. micranthum and G. sokotensis aqueous extracts on blood glucose, cholesterol, and triglyceride.

	Blood glucose (g/L)	Cholesterol (mmol/L)	Triglycerides (mmol/L)
Control	1.31 ± 0.06	1.91 ± 0.03	0.31 ± 0.01
CMAE 100 mg/kg	0.75 ± 0.02	0.93 ± 0.07^***^	0.27 ± 0.00
GSAE 100 mg/kg	0.77 ± 0.01	0.53 ± 0.17^***^	0.30 ± 0.01
CMAE 200 mg/kg	0.95 ± 0.01	1.17 ± 0.02^***^	0.24 ± 0.04^***^
GSAE 200 mg/kg	0.95 ± 0.01	1.05 ± 0.26^***^	0.20 ± 0.01^***^
CMAE 400 mg/kg	1.64 ± 0.01	1.29 ± 0.13^***^	0.23 ± 0.02^***^
GSAE 400 mg/kg	1.54 ± 0.05	1.26 ± 0.16^***^	0.10 ± 0.00^***^
Satellite control	0.99 ± 0.03	1.74 ± 0.03	0.31 ± 0.02
Satellite CMAE 400 mg/kg	1.00 ± 0.02	1.18 ± 0.07^**###**^	0.28 ± 0.02
Satellite GSAE 400 mg/kg	0.97 ± 0.04	1.72 ± 0.35^**###**^	0.30 ± 0.00

Changes in blood glucose, triglycerides, and cholesterol levels measured after 90 days of C. micranthum (CMAE) and G. sokotensis (GSAE) aqueous extracts administration and during a 14-day recovery period. Data are expressed as Mean ± SEM (n = 5), *** p < 0.0001, compared to the control group, ^**###**^ p < 0.0001, compared to the satellite control group.

#### 3.4.6 Effect of *C. micranthum* and *G. sokotensis* aqueous extracts on hematological parameters

*C. micranthum* aqueous extract at all doses resulted in no significant (p > 0.05) changes in globulins and blood cells compared to the control (Table VIII).

**Table VIII:** Effect of C. micranthum aqueous extract on some hematological parameters.

	Control	CMAE 100 mg/kg	CMAE 200 mg/kg	CMAE 400 mg/kg	Satellite control	Satellite 400 mg/kg
Hemoglobin (g/dL)	15.38 ± 0.66	14.74 ± 0.43	14.70 ± 0.38	15.29 ± 0.36	15.58 ± 0.73	15.53 ± 0.06
White blood cells (10^3^/µL)	4.56 ± 0.04	4.92 ± 0.03	4.68 ± 0.10	4.70 ± 0.14	4.64 ± 0.15	4.78 ± 0.12
Red blood cells (10^3^/µL)	8.84 ± 0.29	8.60 ± 0.25	8.66 ± 0.07	8.75 ± 0.19	8.93 ± 0.18	8.72 ± 0.22
Platelets (10^3^/µL)	1018.40 ± 3.05	1023.80 ± 2.59	1017.00 ± 2.24	1015.40 ± 3.36	1000.60 ± 7.60	959.40 ± 11.59
Lymphocyte(10^3^/µL)	2.68 ± 0.08	2.79 ± 0.03	2.53 ± 0.11	2.50 ± 0.12	2.82 ± 0.09	2.86 ± 0.11
Monocytes (10^3^/µL)	0.05 ± 0.01	0.07 ± 0.01	0.07 ± 0.02	0.04 ± 0.01	0.08 ± 0.03	0.04 ± 0.01

Changes in some hematological parameters in rats after taking *C. micranthum* aqueous extract (CMAE) for 90 days and during a 14-day recovery period. Data are expressed as Mean ± SEM (n = 5), p > 0.05, compared to the control group. Satellite compared to the satellite control.

Blood counts revealed no significant (p > 0.05) difference between the cells in GSAE and those in the control group (Table IX).

**Table IX:** Effects of G. Sokotensis extract on some hematological parameters.

	Control	GSAE 100 mg/kg	GSAE 200 mg/kg	GSAE 400 mg/kg	Satellite control	Satellite
Red blood cells (10^3^/µL)	8.80 ± 0.29	8.74 ± 0.13	8.55 ± 0.23	8.57 ± 0.09	8.93 ± 0.18	8.46 ± 0.25
Hemoglobin (g/dL)	15.38 ± 0.66	15.46 ± 0.22	15.48 ± 0.13	15.42 ± 0.84	15.58 ± 0.73	15.04 ± 0.43
White blood cells (10^3^/µL)	4.56 ± 0.04	5.11 ± 0.23	5.26 ± 0.09	4.64 ± 0.13	4.64 ± 0.15	4.85 ± 0.08
Lymphocytes(10^3^/µL)	2.68 ± 0.08	2.70 ± 0.22	2.95 ± 0.10	2.44 ± 0.19	2.82 ± 0.09	2.91 ± 0.07
Monocytes (10^3^/µL)	0.05 ± 0.01	0.06 ± 0.02	0.06 ± 0.01	0.07 ± 0.02	0.08 ± 0.03	0.07 ± 0.03
Platelets (10^3^/µL)	1018.40 ± 3.05	1164.00 ± 10.40	1170.40 ± 8.29	1160.20 ± 15.42	1000.60 ± 7.60	1030.80 ± 11.90

Changes in some hematological parameters in rats after taking *G. sokotensis* aqueous extract (GSAE) for 90 days and during a 14-day recovery period. Data are expressed as Mean ± SEM (n = 5), p > 0.05, compared to the control group.

## 4 Discussion

Many medicinal herbs have been used as a therapeutic source in treating various human ailments throughout the world [32]. According to the World Health Organization, approximately 80 % of the population worldwide depends on herbal medicine for their healthcare needs, especially in rural areas. The lack of proper knowledge and awareness regarding their chemical composition, safety dose, and toxicity led this study to inventory the medicinal plants used by traditional practitioners to treat osteoporosis and related pathologies, and to assess the safety of *C. micranthum* and *G. sokotensis* aqueous extracts through appropriate phytochemical screening and *in vivo* approaches. The survey results show a predominance of men. These results are in agreement with others on the knowledge of medicinal plants from Burkina Faso used to increase physical performance [13]. Men have greater freedom of choice and exercise than women [33]. In some cultures, inheritance is more transmitted to men. However, they contradict those who found a predominance of women (61.20 %) in their study in Niger on the knowledge and use of *C. micranthum* [33]. The interest of old people in traditional medicine may be because they are more conservative and traditionalist. It’s also argued that young people are less interested in traditional practices. The complexity of the field of traditional medicine could be a reason. The various plants mentioned are divided into 33 families, making up a range of plants to combat osteoporosis and related diseases. Several of these plants have already been reported to manage osteoporosis and related diseases [34]. Each part of the plant has some metabolites which are important in health care [35]. *G. sokotensis* and *C. micranthum*, as in some studies, were the most frequently cited by traditional practitioners [31]. This led to identifying their components and evaluating their safety.

Toxicity studies serve as a preclinical review and help minimize time and costs in clinical trials [36]. The administration of a single dose of *C. micranthum* and *G. sokotensis* aqueous extracts (2000 mg/kg) to mice did not induce any visible signs of toxicity either during the first 4 h of administration or during the 14 days of experimentation. All the animals were found alive at the end of the experimental period, which suggests that the lethal dose 50 (LD50) of the aqueous extract is above 2000 mg/kg. The result of the median lethal dose of the extract coincides with the work of Fornari et al. (2014), where no mortality was observed with the dose of 2400 mg/kg of *G. sokotensis* ethanolic extract. The studies of Amali showed no symptoms and no toxic reactions of *C. micranthum* extract at the dose of 2000 mg/kg bw [37]. According to the OECD’s Globally Harmonised System of Classification and Labelling of Chemicals, *C. micranthum* and *G. sokotensis* are considered practically non-toxic and classified as category 5 [38].

Since no toxic effects were observed in the acute study, an additional study was conducted to assess the subchronic toxicity [27] of *C. micranthum* and *G. sokotensis* aqueous extracts during a 90-day experiment on rats. Body-weight gain and relative weight of organs serve as good indicators of health status, including physiological and pathological conditions of experimental animals [39]. Any parameter alteration indicates vital signs for toxicity. In the present study, no significant change was observed in the relative weight of organs (heart, liver, spleen, kidney) of all the rats. These results may then confirm the low toxicity of both aqueous extracts.

To evaluate the toxicity of any new compound, it is essential to know the state of vital organs kidneys and liver, which can be verified by biochemical estimation [40]. Serum creatinine, as a more precise indicator of renal function than urea, is instrumental in determining potential kidney dysfunction [41]. Elevated serum creatinine levels or inefficient clearance can signify irregular glomerular filtration rates, which could potentially lead to acute kidney injury. Furthermore, urea increases in conditions where renal clearance decreases, indicating acute or chronic renal failure. The absence of significant changes observed in the aforementioned indices implies that the administration of the extracts did not exhibit any nephrotoxicity or influence the function of the kidneys. Nephroprotective effects are mainly mediated by reducing oxidative stress and inflammatory response [42]. Flavonoids are a family of polyphenol antioxidants that are effective in combating high levels of oxidative stress and could protect the kidneys [43]. They are distinguished by the presence of multiple phenol rings, C to C double bonds, and hydroxyl groups [44]. These structural characteristics confer the antioxidant function of flavonoids, and the number and location of hydroxyl groups influence the biological activity of the flavonoids [45]. Their capabilities, such as scavenging free radicals, donating hydrogen atoms, and chelating metal cations [46], make them potential antioxidants. With combined anti-inflammatory and antioxidant capacities, flavonoids are the most promising agents for the treatment of acute ischemic kidney injury [47] and protective effects against oxidative damage in the liver of streptozotocin-cadmium-induced diabetic model [48]. Transaminases (ASAT, ALAT) are the main indicators for evaluating liver function and the response to liver injury [49]. A low level of liver enzymes (AST and ALT) indicates a hepato-protective effect of the plant [50], which could explain the results observed. The amount of total cholesterol, triglycerides was determined to evaluate the effect of the extract on the lipid profile. There are correlations between dyslipidaemia, hypertension, and atherosclerosis, on the one hand, and abnormalities in bone metabolism, on the other [51]. High triglyceride and cholesterol levels can affect pro-inflammatory cytokines, such as tumour necrosis factor alpha and interleukin-6, and lead to bone alteration [52]. The decrease in lipid could be due to the terpenoids’ effects [53]. Terpenes seem to regulate lipid metabolism through several mechanisms, including inhibition of hepatic lipid accumulation, regulation of steatosis, inhibition of hepatic lipogenesis, and promotion of fatty acid oxidation via modulation of key enzymes and genes [54]. The mechanism of action of terpenes includes stimulation of insulin secretion by blocking K^+^-adenosine triphosphate (ATP)-dependent channels in the membranes of pancreatic β-cells, causing depolarisation in the plasma membrane and consequently opening calcium channels and promoting the secretion of insulin vesicles into the bloodstream [55]. Blood parameter analysis is important for risk assessment because changes in the predictive value for human toxicity are important [56]. The non-significant change in hematological parameters explains why no sign of inflammation was observed in the structure of all investigated organs, suggesting a minor cause of the above difference in blood cells. The extracts of *C. micranthum* and *G. sokotensis* could be used to treat a wide range of pathologies without major side effects. Their usage as a drug can also be confirmed by preclinical studies.

## 5 Conclusion

This study illustrates a diversity of plant species used by traditional practitioners in the fight against osteoporosis and related diseases. This range of plants reflects the diversity of knowledge across different regions. The ageing appearance of traditional practitioners encourages us to multiply studies in this direction to preserve their knowledge. Phytochemical screening revealed the presence of a wide range of compounds, including flavonoids. The acute oral toxicity study showed that the aqueous extracts of *C. micranthum* and *G. sokotensis* were considered non-toxic up to a dose of 2000 mg/kg bw upon single-day exposure. The safety dose evaluation of the extracts following their regular consumption for an extended period (90 days) was also considered safe. It confirmed the safety dose evaluation and non-toxic nature of the aqueous extracts of *C. micranthum* and *G. sokotensis,* but prevented the risk they could present at high doses. This study contributes to the validation of the plant’s ethnobotanical use and potentially contributes to drug development for some diseases.

## CRediT authorship contribution statement

**YOUGBARE Wendyam Joëlle Raymonde:** Writing – review & editing, Writing – original draft. **TINDANO Basile:** Writing – original draft, Validation, Supervision, Project administration, Methodology. **BAYALA Balé:** Validation, Resources, Project administration. **OWONA Pascal Emmanuel:** Formal analysis, Data curation. **ZABRE Généviève:** Funding acquisition, Data curation. **OUEDRAOGO Elisabeth:** Writing – review & editing, Writing – original draft, Visualization, Software, Methodology, Investigation, Formal analysis.

## Declaration of Competing Interest

The authors declare the following financial interests/personal relationships which may be considered as potential competing interests: Elisabeth OUEDRAOGO reports financial support was provided by FONRID. If there are other authors, they declare that they have no known competing financial interests or personal relationships that could have appeared to influence the work reported in this paper

## Data Availability

Data will be made available on request.
